# A Bureau of Information

**Published:** 1891-08-08

**Authors:** 


### Editor's Letter Box.

### A BUREAU OF INFORMATION.

Mr. P. Michelli, Hon. Secretary of the Hospitals Association, 140, Strand, W.C., writes:

Will you allow me to call attention to the Inquiry Bureau of this association, which has now been in active operation for some time, and which has, I believe, afforded much valuable information in a variety of matters affecting hospital administration, &c.? Upon the value of knowing where to make inquiry it is unnecessary for me to comment. I will only add that the council of the association invite either members or non-members to make use of this source of information, believing that it will prove of much advantage and convenience to hospital managers and the public generally.

